# Immunometabolic regulation of disulfidptosis in orthopedic diseases: mechanistic heterogeneity and therapeutic targets

**DOI:** 10.3389/fimmu.2025.1647931

**Published:** 2025-10-28

**Authors:** Xiaoming Zhao, Chen Zhang, Lian Qu, Jun Gao, Shaobo Wu, Yilei Zhang, Yingang Zhang

**Affiliations:** ^1^ Department of Orthopaedics of the First Affiliated Hospital, Xi’an Jiaotong University, Xi’an, Shaanxi, China; ^2^ The Institute of Molecular and Translational Medicine, Department of Biochemistry and Molecular Biology, School of Basic Medical Sciences, Xi’an Jiaotong University Health Science Center, Xi’an, Shaanxi, China

**Keywords:** disulfidptosis, bone diseases, oxidative stress, immunometabolism, targeted therapy

## Abstract

Disulfidptosis is a novel form of programmed cell death triggered by cystine metabolic disorders and disulfide stress, initially studied primarily in the context of tumors. In recent years, its role in the occurrence and development of orthopedic diseases has gained increasing attention. This review systematically explores the dual regulatory mechanisms of disulfidptosis in degenerative orthopedic diseases, such as intervertebral disc degeneration, osteoporosis, and osteoarthritis, as well as in malignant bone tumors like osteosarcoma, along with their immunometabolic basis. The research findings indicate that in degenerative lesions, microenvironmental stresses such as ischemia and hypoxia exacerbate tissue degeneration by promoting abnormal accumulation of disulfide bonds and damaging the cytoskeleton. In osteosarcoma, tumor-associated oxidative stress can induce metabolism-dependent cell death, providing new opportunities for targeted therapy. The article further summarizes key signaling pathways and molecular regulatory networks, discussing the potential value of targeted intervention strategies in slowing disease progression and achieving precision treatment.

## 1 Introduction

In recent years, the field of programmed cell death has witnessed a breakthrough with the formal definition of disulfidptosis as a novel form of cell death. In 2023, Liu et al. (1) first revealed this new phenomenon: when cells with high expression of the cystine transporter SLC7A11 encounter glucose deprivation, the regeneration of NADPH is hindered, leading to an imbalance in cystine reduction. The abnormal accumulation of disulfides induces cross-linking and contraction of cytoskeletal proteins, ultimately resulting in unique cellular disintegration. This discovery not only enriches the theoretical framework of cell death but also opens new perspectives for studying the pathological mechanisms of orthopedic degenerative diseases and bone tumors, due to the close association of disulfidptosis with metabolic stress and oxidative damage in various diseases (2–10), ​​and its potential to modulate immune responses in the bone microenvironment (10–14).

Within the spectrum of orthopedic diseases, degenerative conditions (such as intervertebral disc degeneration, osteoporosis, and osteoarthritis) and malignant bone tumors exhibit distinct pathologies, yet they share the core feature of metabolic microenvironment imbalance. The former is caused by nutritional supply disruptions or age-related oxidative stress, leading to homeostatic disturbances in chondrocytes, osteoblasts, and nucleus pulposus cells (15–17). In contrast, the latter maintains malignant proliferation and chemotherapy resistance through tumor cell metabolic reprogramming pathways (18–21). Notably, the triggering conditions for disulfidptosis (glucose deprivation and high expression of SLC7A11) significantly overlap with the microenvironment of orthopedic diseases: regions of degenerative changes are often accompanied by local ischemia and hypoxia (22), while osteosarcoma cells commonly exhibit abnormal activation of SLC7A11 to cope with oxidative stress (23). This intersection of pathological features suggests that disulfidptosis may play a “double-edged sword” role in the progression of orthopedic diseases—potentially accelerating the death of normal cells, leading to tissue degeneration, while also being strategically induced to eliminate malignant cells.

This review systematically analyzes the bidirectional regulatory role of disulfidptosis in orthopedic diseases for the first time: on one hand, in degenerative conditions such as intervertebral disc degeneration and osteoporosis, the nutrient-deprived microenvironment may exacerbate cell loss by activating the disulfidptosis pathway; on the other hand, the dependency of malignant tumors like osteosarcoma on SLC7A11 can be transformed into a therapeutic target, enabling selective killing through the induction of disulfidptosis. By integrating recent basic research with preclinical data, this article aims to reveal the key molecular switches involved in disulfidptosis during disease progression and assess the translational potential of targeted intervention strategies with immunomodulatory properties, providing a theoretical framework for precision medicine in orthopedic diseases.

## 2 Core molecular mechanisms of disulfidptosis

**Figure 1:**
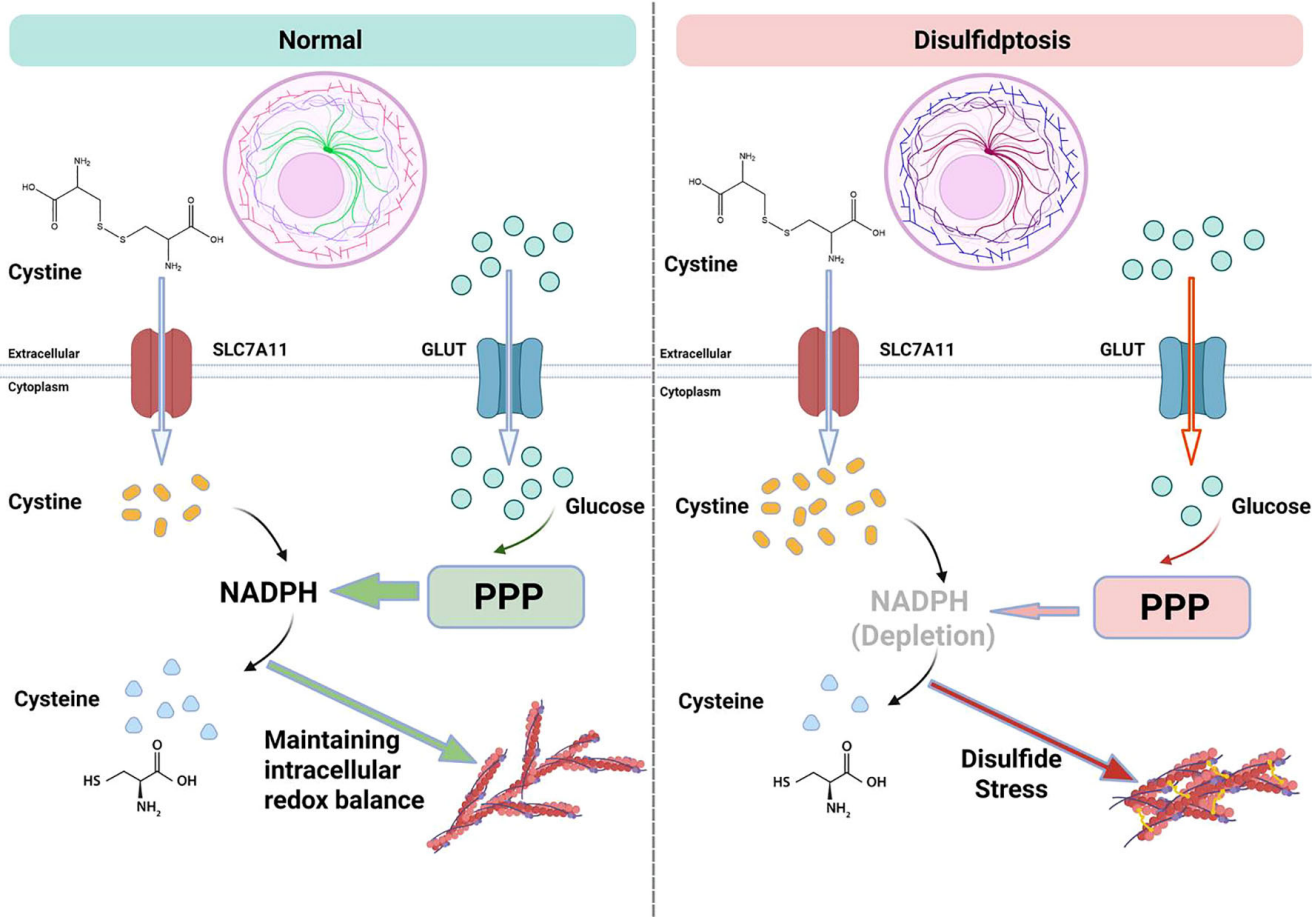
Schematic diagram of disulfidptosis mechanism.

Disulfidptosis is a form of programmed cell death induced by intracellular disulfide stress, with its core mechanism involving cystine metabolism imbalance, abnormal cross-linking of cytoskeletal proteins, and disruption of redox homeostasis (24). Current research has identified its key driving factors and unique characteristics that distinguish it from other forms of programmed cell death, demonstrating mechanistic heterogeneity in both tumor and non-tumor diseases (8, 25). A schematic representation of the mechanisms of disulfidptosis is shown in **Figure 1**.

### 2.1 Pathological associations of disulfidptosis in orthopedic diseases

The pathological relevance of disulfidptosis in orthopedic diseases is rooted in its unique metabolic microenvironment and cellular characteristics. The pathological processes of degenerative orthopedic diseases are often accompanied by local ischemia, hypoxia, nutritional supply disruptions, and age-related oxidative stress. This metabolic stress environment aligns closely with the triggering conditions for disulfidptosis (glucose deprivation and high expression of SLC7A11) (15, 22). Importantly, bone tissue cells (such as chondrocytes and osteoblasts) have a high dependence on cytoskeletal stability, making them particularly sensitive to abnormal disulfide cross-linking. For instance, the integrity of the cytoskeleton in osteoblasts and chondrocytes is crucial for mediating mechanosensitive signaling that regulates their basic functions. Nucleus pulposus cells maintain their morphology against intervertebral pressure through an F-actin network, while osteosarcoma cells rely on cytoskeletal remodeling to achieve invasion and metastasis. This dual vulnerability—metabolic and mechanical—gives the core mechanism of disulfidptosis (cystine metabolism imbalance leading to cytoskeletal collapse) special pathological significance in orthopedic diseases. The following sections will systematically analyze the specific and universal patterns of its molecular mechanisms.

### 2.2 Key driving factors

The initiation of disulfidptosis depends on the functional impairment of the cystine transporter SLC7A11/xCT. SLC7A11 mediates the influx of cystine, contributing to the synthesis of glutathione (GSH) and maintaining cellular antioxidant capacity. However, when cells are in a state of glucose deprivation, the pentose phosphate pathway (PPP) is suppressed, leading to insufficient NADPH production. As a result, cystine cannot be reduced to cysteine and accumulates abnormally within the cell, creating disulfide stress. This process is particularly pronounced in cells with high expression of SLC7A11, such as osteosarcoma cells and nucleus pulposus cells from degenerated intervertebral discs.

The unreduced cystine and its derivatives (such as cysteamine) form abnormal disulfide cross-links with the thiol groups of cytoskeletal proteins, including actin (ACTB) and non-muscle myosin heavy chain (MYH9). This leads to increased rigidity of the cytoskeletal network and loss of contractile function, ultimately resulting in plasma membrane rupture and cellular disintegration. Studies have shown that knocking down SLC7A11 or using disulfide reducing agents can effectively block this cell death process (7). Conversely, the inhibition of thioredoxin reductase activity further exacerbates the reduction deficiency of cystine (26), creating a vicious cycle of “cystine influx-NADPH depletion”.

### 2.3 Differences from other forms of programmed cell death

Disulfidptosis exhibits significant differences in molecular mechanisms and morphological characteristics compared to other forms of programmed cell death (27, 28). While both disulfidptosis and ferroptosis share SLC7A11-mediated cystine metabolism and glutathione (GSH) depletion mechanisms, disulfidptosis does not rely on lipid peroxidation and cannot be blocked by ferroptosis inhibitors, indicating its independence from ferroptosis signaling pathways. In apoptosis and necroptosis, typical events such as caspase activation or MLKL phosphorylation are not involved in the regulation of disulfidptosis. Moreover, disulfidptosis lacks characteristic morphological changes such as apoptotic body formation or plasma membrane pore formation. The uniqueness of disulfidptosis lies in its death signal being directly derived from mechanical damage to the cytoskeleton induced by disulfide stress, a mechanism that has been validated in chondrocytes of osteoarthritis and osteoclast precursor cells in osteoporosis.

### 2.4 Mechanistic heterogeneity in tumor and non-tumor diseases

The regulation of disulfidptosis exhibits essential differences between tumor and non-tumor diseases. In tumors, its triggering often arises from acute metabolic stress induced by therapeutic interventions. Tumor cells, due to high expression of SLC7A11, develop “cystine addiction,” where NADPH depletion leads to reduced cystine metabolism and disulfide-mediated cytoskeletal collapse (29–31). This mechanism is utilized for the targeted killing of malignant cells ​​and may synergize with immunotherapy. In contrast, in non-tumor diseases, chronic microenvironments induce compensatory cystine metabolism imbalance through epigenetic modifications (e.g., NFATc1 activation of SLC7A11), ​​creating conditions that promote pro-inflammatory immune cell infiltration (32–34). Terminally differentiated cells, with limited metabolic plasticity, struggle to cope with sustained NADPH deficiency, ultimately leading to progressive functional loss. Therefore, intervention strategies must accurately differentiate between disease types—tumor treatments should focus on activating disulfidptosis signaling while modulating the immunosuppressive microenvironment​​, while non-tumor diseases require the inhibition of this pathway to maintain cellular homeostasis to restore immune homeostasis.

## 3 Role of disulfidptosis in orthopedic diseases

Disulfidptosis, as a metabolism-dependent form of cell death, exhibits a significant “pro-death - anti-death” bidirectional regulatory characteristic in orthopedic diseases. In bone tumors, excessive activation of disulfidptosis can effectively eliminate malignant cells, while in degenerative diseases, the activation of disulfidptosis exacerbates the functional decline of cells. This bidirectional regulation is closely related to the disruption of the immune metabolic microenvironment. In orthopedic degenerative diseases (such as intervertebral disc degeneration, osteoporosis, osteoarthritis, etc.), chronic metabolic stress (ischemia, hypoxia, oxidative stress) is highly coupled with the key triggering conditions of disulfidptosis through the inflammation factor-metabolic enzyme network, accelerating the functional loss of terminally differentiated cells. Anti-death mechanisms can protect these cells from functional decline. In contrast, in bone tumors (such as osteosarcoma), malignant proliferation relies on cystine metabolic reprogramming and immune evasion, providing a new strategy for targeting and inducing disulfidptosis to eliminate tumor cells. This heterogeneity in immune metabolic regulation results in disulfidptosis displaying entirely opposite pathological effects in orthopedic diseases. The following sections will systematically analyze the specific mechanisms of disulfidptosis in orthopedic diseases through key molecular networks and disease-specific regulatory features (**Table 1**).

**Table 1:** Molecular mechanisms and microenvironmental triggers of disulfidptosis in orthopedic diseases​.

Disease type	Core triggers and microenvironment	Key regulatory molecules and pathways	Downstream effects and pathological outcomes	Current research limitations
​​Intervertebral Disc Degeneration​​	Glucose deprivation, hypoxia (endplate calcification), inflammatory factors (IL-1β, IL-6, TNF-α)	​>​SLC7A11​>​ (upregulated), ​>​GLUT1-4​>​ (downregulated), ​>​FLNA​>​, ​>​MYH9​>​, ​>​ACTB> ​>​Pathways​>​: SLC7A11/NADPH imbalance axis, FN1-CD44 inflammatory interaction axis, Endoplasmic reticulum stress	NADPH depletion → F-actin cytoskeleton abnormal disulfide cross-linking → Nucleus pulposus cell disintegration and death → Extracellular matrix degradation, disc structure destruction	Conclusions largely based on bioinformatics analysis and *in vitro* cell experiments; lack of validation in *in vivo* models.
​​Osteoporosis​​	RANKL stimulation, inadequate nutrient supply, oxidative stress, inflammatory microenvironment (TNF-α, IL-6)	​>​SLC7A11​>​, ​>​RPN1​>​, ​>​NFATc1​>​, ​>​RAC1​>​ ​>​Pathways​>​: NFATc1-SLC7A11-TXNRD1 signaling axis, Immune cell infiltration (monocytes/macrophages)	Osteoclast precursor disulfide stress → Excessive osteoclast differentiation/hyperactivity → Bone resorption and formation imbalance → Bone microstructure destruction	Studies rely heavily on machine learning and single-cell sequencing inferences; lack of direct functional experimental evidence for disulfidptosis in osteoclasts.
​​Osteoarthritis​​	Inflammatory factors (IL-1β, TNF-α, IL-17, TGF-β), oxidative stress, metabolic disturbances	​>​SLC3A2​>​ (downregulated), ​>​NCKAP1​>​, ​>​PDLIM1​>​, ​>​OXSM​>​, ​>​NDUFS1/NDUFC1​>​ ​>​Pathways​>​: Mitochondrial complex I dysfunction, Cytoskeleton-inflammation positive feedback loop	Chondrocyte cysteine uptake impairment​​/metabolic imbalance → Loss of cytoskeletal stability → Chondrocyte death → Cartilage matrix degradation, joint degeneration	Mechanistic studies are fragmented; lack of comprehensive interpretation of the “metabolism-cytoskeleton-immune” cross-regulatory network.
​​Osteosarcoma​​	Glucose deprivation (chemotherapy-induced), hypoxia, tumor oxidative stress microenvironment	​>​SLC7A11​>​ (high expression), ​>​ACTB​>​, ​>​MYH9​>​, ​>​HMGB1​>​, ​>​LRPPRC> ​>​Pathways​>​: SLC7A11-PI3K/Akt/mTOR axis, HMGB1-TLR4-NF-κB axis, m6A-PD-L1 immune escape axis	“Cystine addiction” → NADPH depletion under chemotherapy → Cytoskeleton collapse → ​>​**Induction of tumor cell death​>​​>​Concurrently**​>​: Promotes PD-L1 expression → Immunosuppressive microenvironment	Most mechanistic evidence comes from bioinformatic models and correlative analyses; insufficient validation of the anti-cancer efficacy of targeting disulfidptosis *in vivo*.

### 3.1 Intervertebral disc degeneration

#### 3.1.1 Pathological characteristics and potential association with disulfidptosis

The core pathological features of intervertebral disc degeneration (IVDD) include the degradation of the nucleus pulposus (NP) extracellular matrix, homeostatic imbalance, and disturbances in the nutritional microenvironment (35–40). Due to the avascular nature of intervertebral discs, nutrient supply relies on endplate diffusion, and calcification or aging of the endplates can impede the transport of glucose and oxygen, exacerbating metabolic stress in NP cells (41–43). As the only cellular component within the intervertebral disc, NP cell survival is highly dependent on glucose metabolism to maintain redox homeostasis. Research indicates that glucose deprivation can directly induce NP cell death; however, conventional apoptosis or necrosis inhibitors fail to fully rescue cell survival, suggesting the involvement of a novel cell death mechanism (44). Disulfidptosis, as a newly identified form of programmed cell death, is triggered by disulfide stress resulting from intracellular NADPH depletion and abnormal cross-linking of cytoskeletal proteins. This mechanism aligns closely with the restricted glucose metabolism and redox imbalance observed in IVDD. The high expression of the cystine transporter SLC7A11 in NP cells during degeneration may accelerate cystine uptake, further depleting NADPH reserves and triggering the key pathways of disulfidptosis.

#### 3.1.2 Mechanisms of disulfidptosis in intervertebral disc degeneration

Recent studies have confirmed the role of disulfidptosis in IVDD for the first time (44). (1) SLC7A11-NADPH Imbalance Drives Disulfide Stress: SLC7A11 is significantly upregulated in degenerated nucleus pulposus tissue, promoting excessive uptake of cystine and depleting NADPH as it is reduced to cysteine. Glucose deprivation inhibits the pentose phosphate pathway (PPP), blocking NADPH production and leading to an increased NADP+/NADPH ratio, along with the abnormal accumulation of disulfides in cytoskeletal proteins such as actin (e.g., FLNA, MYH9). This results in cytoskeletal collapse and cell death. (2) Downregulation of Glucose Transporters (GLUTs) Exacerbates Metabolic Crisis: Single-cell sequencing analysis revealed a significant negative correlation between SLC7A11 and SLC2A1-4 (GLUT1-4) in the degenerative chondrocyte subpopulation (C4). The reduced expression of GLUTs further limits glucose influx, amplifying the effects of NADPH depletion. (3) Synergistic Effects of Endoplasmic Reticulum Stress and Oxidative Stress: The degenerative C4 subpopulation is enriched in genes related to endoplasmic reticulum stress, the p53 pathway, and oxidative stress, suggesting that disulfidptosis may accelerate NP cell loss by activating the unfolded protein response (UPR) and pro-apoptotic signals. Experimental evidence shows that the glucose analog 2-DG can reverse NADP+/NADPH imbalance by supplementing NADPH, significantly inhibiting disulfidptosis in NP cells. This provides a new strategy for targeting metabolic reprogramming to intervene in IVDD. Research on intervertebral disc degeneration is currently relatively limited, with verification methods primarily based on bioinformatics analysis and basic cellular experiments. The reliability of the conclusions and the underlying mechanisms require further exploration in the future.

### 3.2 Osteoporosis

#### 3.2.1 Pathological characteristics and potential association with disulfidptosis

Osteoporosis (OP) is characterized by reduced bone density, compromised bone microstructure, and an increased risk of fractures, with the core pathological mechanism involving a dynamic imbalance between bone resorption and formation (45–51). Research indicates that metabolic disorders, immune dysregulation, and subsequent cell death within the bone microenvironment collectively contribute to the pathogenesis of OP (52–58). In recent years, a novel form of cell death—disulfidptosis—has been increasingly recognized for its role in osteoporosis. Disulfidptosis is a form of programmed cell death triggered by the abnormal accumulation of intracellular disulfides, characterized by SLC7A11-mediated excessive cystine uptake in a glucose-deprived environment, leading to NADPH depletion and disulfide stress, ultimately resulting in abnormal cross-linking of cytoskeletal proteins and cell death. The pathological environment of osteoporosis, with its unique microenvironmental characteristics of low glucose and low oxygen partial pressure, provides conditions conducive to the occurrence of disulfidptosis (59, 60). Bone-related cells, such as bone marrow mesenchymal stem cells (BM-MSCs) and osteoclast precursors, exhibit upregulated expression of SLC7A11 and redox imbalance under the stimulation of inflammatory factors, further increasing their susceptibility to disulfidptosis. Notably, the infiltration of immune cells and the release of inflammatory factors (e.g., TNF-α, IL-6) present in the bone microenvironment of osteoporosis patients not only directly participate in the regulation of bone metabolism but may also form a complex interactive network with the occurrence and progression of disulfidptosis by affecting cystine metabolism and redox balance (61, 62). Disulfidptosis may intertwine with traditional pathological mechanisms of osteoporosis, participating in the disease process through a unique “metabolic-immune-cytoskeletal” regulatory axis.

#### 3.2.2 Mechanisms of disulfidptosis in osteoporosis

Recent studies have revealed the significant role of disulfidptosis in the progression of osteoporosis. In terms of metabolic regulation and osteoclast activation, research by Zhong et al. (61) found that disulfidptosis plays a critical role in osteoclast differentiation through the NFATc1-SLC7A11-TXNRD1 signaling axis. The study showed that during RANKL-induced osteoclast differentiation, NFATc1 significantly upregulates the expression of SLC7A11, enhancing the capacity of osteoclast precursors to uptake cystine. When TXNRD1 activity is inhibited, dysregulation of intracellular cystine metabolism leads to abnormal accumulation of disulfides, ultimately triggering characteristic F-actin contraction and cell death. This form of cell death is specific and cannot be reversed by conventional cell death inhibitors but can be effectively intervened by thiol compounds. Animal experiments confirmed that targeting this pathway can significantly improve bone resorption markers and bone microstructure.

In terms of remodeling the immune microenvironment, disulfidptosis participates in the immune metabolic imbalance of osteoporosis by regulating immune cell function. Wang et al. (59) conducted single-cell analysis of clinical data, revealing that osteoporosis patients can be categorized into different disulfidptosis subtypes, with high-scoring subtypes significantly associated with the infiltration of specific immune cell subsets (such as monocytes and T follicular helper cells). These immune cells activate the osteoclast differentiation pathway by secreting pro-inflammatory factors. Additionally, abnormal expression of PGRMC2 has been found to be associated with osteoporosis risk, potentially participating in the regulation of the bone immune microenvironment by influencing the differentiation process of monocytes into macrophages. Genetic analysis further supports the protective role of PGRMC2 in osteoporosis.

In terms of cytoskeletal stability and bone homeostasis, Zhang et al. (60) found that disulfidptosis regulators participate in the imbalance of bone remodeling by influencing cytoskeletal dynamics. Abnormal expression of key cytoskeletal proteins, such as FLNA and ACTB, leads to decreased stability of the actin network, promoting osteoclast differentiation. Conversely, excessive activation of regulators like RAC1 accelerates the formation of osteoclast bone resorption structures. Additionally, research by Pan et al. (62) revealed that abnormal expression of RPN1 recruits specific immune cells through pro-inflammatory signaling pathways, while targeted inhibition of RPN1 can effectively improve the abnormal bone microstructure in animal models.

These three mechanisms are interwoven, collectively forming a complete framework for the involvement of disulfidptosis in the pathological processes of osteoporosis: metabolic reprogramming alters cell fate determination, remodeling of the immune microenvironment affects local inflammatory states, and regulation of cytoskeletal stability directly participates in the modulation of bone resorption function. Nevertheless, the conclusions of this section primarily rely on single-cell sequencing, machine learning, and *in vitro* studies, lacking further validation through *in vivo* experiments and clinical trials, which will be a focus of future research.

### 3.3 Osteoarthritis

#### 3.3.1 Pathological features and potential association with disulfidptosis

Osteoarthritis (OA) is characterized by degeneration of articular cartilage, synovial inflammation, and imbalance in chondrocyte homeostasis (63–68). Chondrocytes are critical for maintaining the function of articular cartilage, and their abnormal death (such as apoptosis and necroptosis) is closely related to the progression of OA (69–75).

Recently discovered disulfidptosis, a novel form of programmed cell death, is triggered by the abnormal accumulation of disulfides within cells, leading to cross-linking of cytoskeletal proteins and collapse of the actin network. This mechanism aligns well with the loss of cytoskeletal stability observed in chondrocytes during OA (76, 77). In the OA microenvironment, oxidative stress, inflammatory factors (such as IL-1β and TNF-α), and abnormalities in glucose metabolism may activate SLC7A11, leading to excessive cystine uptake and exacerbating intracellular disulfide stress, thereby triggering disulfidptosis. Moreover, dysfunction of mitochondrial respiratory chain complexes (such as dysregulation of NDUFS1 and NDUFC1 expression) may further amplify energy metabolism disturbances and redox imbalances, creating a vicious cycle that promotes the occurrence of disulfidptosis.

#### 3.3.2 Mechanisms of disulfidptosis in osteoarthritis

Recent studies utilizing multi-omics analysis have revealed the critical role of disulfidptosis-related genes (DRGs) in osteoarthritis (OA). Wei et al. (76)integrated six OA datasets and single-cell sequencing, discovering that DRGs such as SLC3A2 and NDUFC1 are specifically highly expressed in OA chondrocytes and significantly correlated with the activation of pro-inflammatory pathways like IL-17 and TGF-β. Experimental validation showed that SLC3A2 expression is downregulated in OA models, and its deficiency exacerbates oxidative stress by inhibiting cystine transport, leading to the upregulation of extracellular matrix degradation markers (MMP3, ADAMTS5). This suggests that SLC3A2 may influence OA progression by regulating the balance between disulfidptosis and autophagy.

Cao et al. (77)employed machine learning to identify SLC3A2 and PDLIM1 as core DRGs. They found that PDLIM1 is abnormally highly expressed during late-stage chondrocyte differentiation, disrupting cytoskeletal integrity by competitively binding to α-actinin 4 (ACTN4) while inhibiting autophagy-related pathways (such as the mTOR-ULK1 axis), exacerbating the inflammatory response in chondrocytes. Hu et al. (78) further investigated that OA samples with different disulfidptosis scores exhibit distinct characteristics of immune cell infiltration. The C1 subtype (high score) shows activation of CD8+ T cells and a decrease in M0 macrophages, while the C2 subtype (low score) is characterized by an increase in Th17 cell infiltration and high expression of pro-inflammatory factors (IL-6, TNF-α). The imbalance in the proportion of these immune cell subpopulations is significantly correlated with the expression levels of key regulatory factors of disulfidptosis (such as NCKAP1, OXSM), indicating that disulfidptosis may participate in the inflammatory process of OA by regulating the activation and function of immune cells. These studies collectively suggest that disulfidptosis is involved in the pathological mechanisms of OA through a multidimensional network of “metabolism - oxidative stress - immune regulation,” providing new directions for targeted intervention.

### 3.4 Osteosarcoma

#### 3.4.1 Pathological features and immunometabolic crosstalk in disulfidptosis

Osteosarcoma, as a highly heterogeneous malignant bone tumor, is characterized by rapid proliferation, a tendency for early metastasis, and chemotherapy resistance (79, 80). Disulfidptosis, as a novel form of programmed cell death, involves a core mechanism of cystine metabolic imbalance mediated by SLC7A11, leading to abnormal accumulation of intracellular disulfides, which in turn triggers cross-linking of cytoskeletal proteins and membrane rupture. This process is highly compatible with the oxidative stress microenvironment of osteosarcoma (81): osteosarcoma cells, due to rapid proliferation, often exist in states of hypoxia and nutrient deficiency, potentially exacerbating cystine-dependent metabolic abnormalities through overexpression of SLC7A11, thereby creating conditions that promote disulfidptosis. Additionally, the high expression of actin-related genes (such as ACTB and MYH9) in osteosarcoma tissues may further increase the sensitivity of the cytoskeleton to abnormal disulfides, providing a molecular basis for the occurrence of disulfidptosis.

#### 3.4.2 Mechanisms of disulfidptosis in osteosarcoma

Recent studies have gradually revealed the regulatory networks and clinical significance of disulfidptosis-related genes (DRGs) in osteosarcoma. At the molecular mechanism level, abnormal activation of the SLC7A11-PI3K/Akt/mTOR signaling axis plays a critical role in the progression of osteosarcoma. Research by Xu et al. (82) indicates that SLC7A11 upregulates HK2 expression and glycolytic activity through mTORC1, while activated PI3K promotes the phosphorylation of GSK3β, inhibiting β-catenin degradation, thus forming a positive feedback loop of “cystine metabolism-glycolysis-Wnt signaling.” Clinical data show that patients with high expression of SLC7A11 exhibit increased sensitivity to mTOR inhibitors, providing important evidence for targeted therapy.

The interaction between microenvironmental factors and cytoskeletal stability is also crucial in the regulation of disulfidptosis. Wang et al. (83) found that exogenous HMGB1 upregulates ACTB expression via the TLR4/MyD88/NF-κB pathway, leading to the depolymerization of the F-actin network and disruption of cell membrane stability, accompanied by changes in histone modifications. This finding reveals the bridging role of the HMGB1-ACTB-TLR4 axis in connecting microenvironmental stimuli with cytoskeletal damage, also providing a new target for therapeutic intervention.

In terms of immune regulation, the prognostic model constructed by Chen et al. (84) shows that LRPPRC, as an m6A reader, recognizes a specific sequence in PD-L1 mRNA and enhances its translation efficiency by recruiting YTHDF1, leading to the functional suppression of CD8+ T cells. Additionally, LRPPRC may enhance the stability of PD-L1 mRNA through m6A modification, creating a malignant cycle of immune suppression and metabolic disorder. Meanwhile, MYH9 regulates the secretion of immune factors via the Hippo-YAP pathway, potentially leading to Treg infiltration and NK cell functional suppression, thus forming an immunosuppressive microenvironment. These findings reveal the important role of disulfidptosis-related genes in tumor immune evasion.

## 4 Dual role of disulfidptosis in targeted therapeutic strategies for orthopedic diseases

Previous studies have shown that Targeted therapies for orthopedic diseases exhibit bidirectional regulatory characteristics, necessitating precise differentiation in intervention strategies for tumor versus non-tumor conditions (85–90). Regarding disulfidptosis-targeted therapeutic strategies, in malignant tumors such as osteosarcoma, therapeutic strategies focus on activating disulfidptosis signaling pathways to induce tumor-specific cell death ​​while concurrently overcoming immunosuppressive barriers, and enhance treatment efficacy ​especially when combined with immune checkpoint inhibitors (91–96). Conversely, in degenerative diseases like intervertebral disc degeneration, osteoporosis, and osteoarthritis, it is crucial to inhibit the excessive activation of disulfidptosis pathways to protect cellular functions, ​​and mitigate pro-inflammatory immune responses and delay tissue degeneration (97–102).

**Figure 2:**
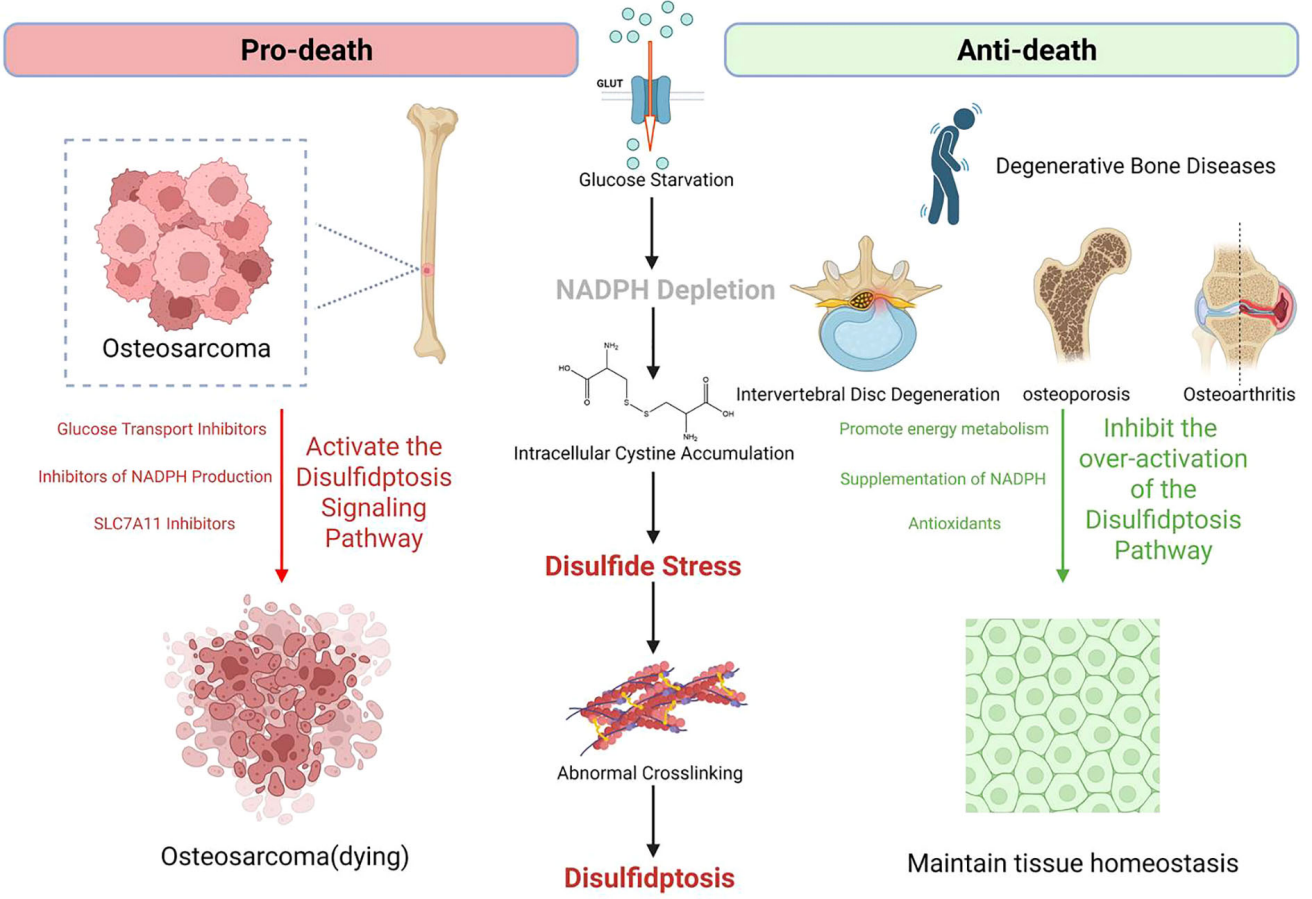
Bidirectional targeting of disulfidptosis in degenerative and malignant orthopedic disorders​.

This “pro-death vs. anti-death” dual logic underscores the core value of targeting disulfidptosis pathways in orthopedic precision medicine with integrated immunomodulation. By tailoring interventions based on the fundamental differences in disease nature, therapeutic goals can be optimized through specific ​​immune-aware molecular mechanisms that simultaneously address metabolic dysfunction and immune microenvironment remodeling. A schematic representation of the “pro-death vs. anti-death” therapeutic strategy is shown in **Figure 2**, and **Table 2** systematically summarizes the main therapeutic principles and disulfidptosis-related precision treatment strategies for the four types of diseases.

**Table 2:** Disulfidptosis-targeted therapeutic strategies for orthopedic diseases​.

Disease​​	​​Strategy & target​​	​​Agents/approaches​​	​​Mechanism​​	​​Challenges & future directions​​
​​IVDD​​	Inhibit SLC7A11 & enhance GLUTs	SLC7A11 inhibitors; GLUT agonists	Reduce cystine overload; improve glucose influx	Lack of specific inhibitors; need localized delivery → Develop disc-penetrating nanocarriers
	Supplement NADPH	2-DG analogs	Activate PPP to boost NADPH	Off-target toxicity → Synthesize novel NADPH precursors
	Modulate ER stress	IRE1α inhibitors	Alleviate UPR-mediated apoptosis	Preliminary crosstalk evidence → Explore combination therapies
​​Osteoporosis​​	Target NFATc1-SLC7A11-TXNRD1 axis	Auranofin, TRi-1	Induce disulfidptosis in osteoclast precursors	Systemic toxicity → Develop bone-targeted nano-formulations
	Inhibit RPN1	Kaempferol	Disrupt pro-disulfidptosis function	Low bioavailability; mechanistic clarity → Optimize derivatives; validate RPN1-ceRNA network
	Regulate immune microenvironment (PGRMC2)	PGRMC2 agonists (to be developed)	Modulate monocyte-macrophage differentiation	No specific agonists available → High-throughput screening for agonists
​​Osteoarthritis​​	Upregulate SLC3A2 & modulate metabolism	SLC3A2 agonists; mTOR inhibitors	Restore cystine/GSH homeostasis	Disease heterogeneity → Apply based on immune subtypes (e.g., C1/C2)
	Target cytoskeleton-inflammation loop (PDLIM1)	PDLIM1 siRNA; inhibitors	Stabilize cytoskeleton; reduce IL-6/MMP13	Delivery challenges → Develop intelligent responsive intra-articular delivery systems
	Immunomodulation based on subtypes	PPM1F inhibitors; IL-17 antagonists	Tailor therapy to immune features	Complex personalized therapy → Establish clear biomarkers for stratification
​​Osteosarcoma​​	Induce disulfidptosis via metabolic stress	Glucose deprivation; SLC7A11 inhibitors	Trigger NADPH depletion and cytoskeleton collapse	TME complexity → Develop strategies to specifically starve tumor cells
	Target cytoskeleton directly (ACTB/MYH9)	HMGB1-TLR4 axis inhibitors	Disrupt actin polymerization	Off-tumor effects → Investigate tumor-specific cytoskeleton vulnerabilities
	Combine with immunotherapy	PD-1/CTLA-4 inhibitors + inducers	Enhance immunogenicity; reverse immunosuppression	Dosing/sequencing unknown → Preclinical studies in immunocompetent PDX models

### 4.1 Intervertebral disc degeneration

In the targeted therapeutic strategies for disulfidptosis in intervertebral disc degeneration (IVDD), research focuses on regulating SLC7A11-mediated redox imbalance to maintain cellular homeostasis. Literature indicates (44)that glucose deficiency in IVDD drives NADPH depletion by upregulating SLC7A11, leading to disulfidptosis. The key to inhibiting this pathway lies in restoring NADPH balance. Experimental evidence shows that 2-deoxy-D-glucose (2-DG) can activate the pentose phosphate pathway to replenish NADPH, significantly reducing the NADP+/NADPH ratio and rescuing cells from glucose deprivation-induced death. Additionally, inhibiting SLC7A11 or enhancing the function of glucose transporters (such as GLUT1/SLC2A1) can block excessive cystine uptake and subsequent oxidative stress. Modulating endoplasmic reticulum stress pathways (such as the unfolded protein response) may also synergistically alleviate disulfidptosis. These strategies provide potential therapeutic targets for delaying intervertebral disc degeneration by precisely inhibiting disulfidptosis signals, such as developing SLC7A11 inhibitors or GLUT agonists to maintain redox homeostasis in nucleus pulposus cells.

### 4.2 Osteoporosis

In targeted therapeutic strategies for disulfidptosis in osteoporosis, research focuses on the precise intervention of key regulatory factors. Zhong et al. (61)found that NFATc1 activates SLC7A11 transcriptionally, driving osteoclast precursor cells’ sensitivity to thioredoxin reductase 1 (TXNRD1) inhibitors (such as Auranofin). Inhibiting TXNRD1 activity leads to cystine accumulation and disulfidptosis mediated by F-actin contraction, significantly reducing bone resorption.

Pan et al. (62)revealed that kaempferol could target and inhibit the aberrantly high expression of disulfidptosis-related gene RPN1, reversing bone microstructure damage in ovariectomized rats, suggesting the potential therapeutic value of flavonoids in regulating RPN1. Wang et al. (33) further discovered that PGRMC2 influences bone metabolism by promoting the differentiation of monocytes into macrophages; its downregulation exacerbates osteoporosis progression, while restoring PGRMC2 levels can improve bone homeostasis. Zhang et al. (60) constructed a diagnostic model based on genes like SLC7A11 and RAC1, proposing a combination therapy that involves regulating the immune microenvironment of monocyte infiltration to inhibit disulfidptosis and maintain the osteoblast/osteoclast balance. These studies provide molecular mechanisms and translational evidence for developing precision treatments targeting disulfidptosis in osteoporosis.

### 4.3 Osteoarthritis

Recent research advancements have led to breakthrough findings in targeted therapeutic strategies for disulfidptosis in osteoarthritis (OA). Wei et al. (76) integrated single-cell sequencing with machine learning algorithms, discovering that SLC3A2 is significantly downregulated in the EC subpopulation of OA chondrocytes. Its deficiency exacerbates cartilage degeneration by activating IL-17 and TGF-β inflammatory pathways. *In vivo* experiments confirmed that upregulating SLC3A2 effectively inhibits disulfidptosis-related F-actin network collapse.

Cao et al. (77) further revealed a bidirectional regulatory mechanism between SLC3A2 and PDLIM1: SLC3A2 maintains redox balance by promoting the cystine/glutathione axis, while aberrant high expression of PDLIM1 in late-stage OA disrupts the autophagy-cytoskeleton balance. Silencing PDLIM1 with siRNA resulted in a 47.3% reduction in inflammatory factors IL-6 and MMP13. Notably, Hu et al. (78) constructed a multi-omics diagnostic model showing that the NCKAP1-OXSM-SLC3A2 regulatory network is closely related to the OA immune microenvironment. Targeted inhibition of PPM1F (a magnesium-dependent phosphatase) increased chondrocyte survival by 32%, with mechanisms involving the restoration of mitochondrial complex I function and reduction of abnormal disulfide accumulation (p< 0.01). These findings provide a theoretical basis for developing specific small molecule inhibitors of disulfidptosis, such as SLC3A2 agonists or PDLIM1 antagonists.

### 4.4 Osteosarcoma

In the research on targeted therapeutic strategies for disulfidptosis in osteosarcoma, multiple studies have revealed key regulatory targets and potential therapeutic directions. Xu et al. (82) found that osteosarcoma cells with high expression of SLC7A11 are prone to disulfidptosis under glucose deprivation, suggesting that inhibiting glucose transport or pharmacologically targeting the SLC7A11 pathway could selectively induce tumor cell death.

Wang et al. (83) utilized single-cell sequencing to discover that HMGB1 regulates ACTB expression and participates in the disulfidptosis process. They confirmed that silencing ACTB significantly reduces osteosarcoma cell viability, while exogenous HMGB1 treatment enhances cell death sensitivity through the TP53/NF-κB signaling axis. Chen et al. (84) constructed a prognostic model indicating that MYH9 and LRPPRC are key risk genes, with their inhibitors potentially activating the disulfidptosis pathway by disrupting cytoskeletal stability. Notably, Zhang et al. (81) pointed out that the activation of disulfidptosis is associated with the remodeling of the immune microenvironment, where patients in the low-risk group demonstrated significantly increased sensitivity to PD-1/CTLA-4 inhibitors. This suggests that combined immune checkpoint blockade may produce a synergistic anti-tumor effect, with drug sensitivity analysis identifying targeted agents such as lapatinib, bortezomib, fruquintinib, and MG-132. Currently, targeted strategies primarily focus on metabolic interventions, key gene regulation, and the development of cytoskeletal-targeting drugs. These avenues provide a multidimensional therapeutic framework for precisely activating disulfidptosis in osteosarcoma.

## 5 Conclusion and outlook

This study systematically reveals the key mechanisms of disulfidptosis in orthopedic diseases and its potential for clinical translation. By integrating multi-omics data and experimental validation, we found that disulfidptosis exhibits distinctly different regulatory patterns in degenerative diseases such as intervertebral disc degeneration and osteoporosis compared to malignant tumors like osteosarcoma. In degenerative diseases, SLC7A11-mediated cystine metabolic imbalance leads to NADPH depletion, triggering abnormal cross-linking of cytoskeletal proteins and cell death; whereas in malignant tumors, targeted activation of the disulfidptosis pathway can selectively kill tumor cells. This finding provides new molecular targets and therapeutic strategies for precision diagnosis and treatment of orthopedic diseases.

Despite the significant progress made in this study, there are still several areas that require improvement: First, the limitations in sample sources may affect the generalizability of the research conclusions. The current study is primarily based on public databases and a limited number of clinical samples (44, 59, 76), and future efforts should expand the sample size and include a broader range of ethnic groups to validate the reliability of the results. Second, regarding mechanism studies, the interactions between disulfidptosis and other forms of programmed cell death, such as apoptosis, ferroptosis, autophagy, and necroptosis, have not been fully elucidated (103–107). In particular, the “molecular switch” function of SLC7A11 in different death pathways requires further exploration. Additionally, the lack of animal models limits the depth of *in vivo* validation, necessitating the development of genetically engineered animal models that more closely resemble human disease characteristics.

Future research should focus on several key directions: In translational medicine, there is an urgent need to develop a ctDNA-based LRPPRC mutation monitoring panel and an imaging radiomics early warning system, while optimizing the drug delivery technology of pH-responsive nanoparticles to improve targeted delivery efficiency. Clinical translational studies should establish SLC7A11 conditional knockout animal models and humanized PDX models to provide a reliable platform for treatment assessment. Mechanistic studies should concentrate on elucidating the spatiotemporal specificity of NFATc1-SLC7A11 transcriptional regulation and clarifying the molecular switch involved in TXNRD1 inhibitor-induced cytoskeletal remodeling. Additionally, exploring the metabolic dialogue mechanisms in the bone marrow microenvironment and developing disulfidptosis-inducing strategies targeting the tumor metabolic microenvironment will provide new insights for achieving precision therapy.
